# Revisiting multi-omics-based predictors of the plasma triglyceride response to an omega-3 fatty acid supplementation

**DOI:** 10.3389/fnut.2024.1327863

**Published:** 2024-02-13

**Authors:** Josiane Morin-Bernier, Juan de Toro-Martín, Valentin Barbe, Rodrigo San-Cristobal, Simone Lemieux, Iwona Rudkowska, Patrick Couture, Olivier Barbier, Marie-Claude Vohl

**Affiliations:** ^1^Centre Nutrition, santé et société (NUTRISS)—Institut sur la nutrition et les aliments fonctionnels (INAF), Université Laval, Québec, QC, Canada; ^2^School of Nutrition, Université Laval, Québec, QC, Canada; ^3^Laboratory of Molecular Pharmacology, Endocrinology and Nephrology Unit, Centre hospitalier universitaire de Québec-Université Laval Research Center, Québec, QC, Canada; ^4^Department of Kinesiology, Université Laval, Québec, QC, Canada; ^5^Faculty of Pharmacy, Université Laval, Québec, QC, Canada

**Keywords:** short-chain fatty acids, bile acids, gut microbiota, metabolic health, metabolomics, precision nutrition

## Abstract

**Background:**

The aim of the present study was to identify the metabolomic signature of responders and non-responders to an omega-3 fatty acid (n-3 FA) supplementation, and to test the ability of a multi-omics classifier combining genomic, lipidomic, and metabolomic features to discriminate plasma triglyceride (TG) response phenotypes.

**Methods:**

A total of 208 participants of the Fatty Acid Sensor (FAS). Study took 5 g per day of fish oil, providing 1.9–2.2 g eicosapentaenoic acid (EPA) and 1.1 g docosahexaenoic (DHA) daily over a 6-week period, and were further divided into two subgroups: responders and non-responders, according to the change in plasma TG levels after the supplementation. Changes in plasma levels of 6 short-chain fatty acids (SCFA) and 25 bile acids (BA) during the intervention were compared between subgroups using a linear mixed model, and the impact of SCFAs and BAs on the TG response was tested in a mediation analysis. Genotyping was conducted using the Illumina Human Omni-5 Quad BeadChip. Mass spectrometry was used to quantify plasma TG and cholesterol esters levels, as well as plasma SCFA and BA levels. A classifier was developed and tested within the DIABLO framework, which implements a partial least squares-discriminant analysis to multi-omics analysis. Different classifiers were developed by combining data from genomics, lipidomics, and metabolomics.

**Results:**

Plasma levels of none of the SCFAs or BAs measured before and after the n-3 FA supplementation were significantly different between responders and non-responders. SCFAs but not BAs were marginally relevant in the classification of plasma TG responses. A classifier built by adding plasma SCFAs and lipidomic layers to genomic data was able to even the accuracy of 85% shown by the genomic predictor alone.

**Conclusion:**

These results inform on the marginal relevance of SCFA and BA plasma levels as surrogate measures of gut microbiome in the assessment of the interindividual variability observed in the plasma TG response to an n-3 FA supplementation. Genomic data still represent the best predictor of plasma TG response, and the inclusion of metabolomic data added little to the ability to discriminate the plasma TG response phenotypes.

## 1 Introduction

The beneficial effects of marine omega-3 fatty acids (n-3 FA) on reducing plasma triglyceride (TG) levels are widely recognized (1), but subject to a large interindividual variability (2). Accordingly, their potential effects in reducing the incidence of cardiovascular disease are still under debate (3, 4). Current recommendations for marine n-3 FA supplementation may therefore be limited by being too general and not addressing the specific needs of certain population subgroups (5). Thus, not all individuals seem to benefit equally from n-3 FA supplementation, with some individuals being relatively insensitive, while others being quite responsive, as extensively reported in the past (2). Indeed, although a reduction of the risk of cardiovascular events by decreasing plasma TG levels has been associated with the consumption of n-3 FA (6), a large heterogeneity in the response to n-3 FA supplementation has also been reported (2). In the Fatty Acid Sensor (FAS) Study, we previously reported that about 30% of individuals do not decrease their plasma TG levels in response to a 6-week n-3 FA supplementation (7).

There is evidence that this interindividual variability is, at least in part, determined by genetic factors, as previously shown in the FAS Study, where a number of single-nucleotide polymorphisms (SNPs) were associated with the plasma TG response in a genome-wide association study (GWAS) (8). After fine mapping and densification of genetic markers by imputation, a genetic risk score (GRS) composed of 31 SNPs was built, explaining more than 49% of the variance of plasma TG levels during the supplementation (9). Finally, lipidomic features in the form of plasma TG species were added to the GRS in a classification tool that was able to correctly identify TG response phenotypes with the accuracy of 75% in a subset of the FAS population (10). Although the combination of genetic information and lipidomic data allowed to predict the plasma TG response to an n-3 FA supplementation with high accuracy, a non-negligible portion of this response remained unexplained. Given the mounting evidence linking gut microbiota to the heterogeneity in the response to nutrients, and specifically, to n-3 FA consumption (11), we hypothesized that part of this unexplained variance may be due to a distinct impact of n-3 FA on the gut microbiota composition of responders vs. non-responders. Since we lacked direct metagenomic data in the FAS Study, we were interested in analyzing the ability of short-chain fatty acids (SCFA) and bile acids (BA) plasma profiles, two well-known gut microbiota-derived metabolites, to aid in the prediction of the plasma TG response to the n-3 FA supplementation. On one hand, significant shifts in the composition of the gut microbiome with a specific increase in SCFA-producing bacteria have been found following an n-3 supplementation (12) and, in the other hand, BAs are further metabolized by the gut microbiota (11), and partly dependent on n-3 FA consumption (13). Recent evidence linking gut microbiota and microbiota-related metabolites with the development of hyperlipidemia (14) and the advances in metabolomic technologies triggered the exploration of the relationship between circulating metabolites, such as SCFA and BA, and cardiometabolic disease risk factors (15). SCFAs appear to be promising metabolites in the modulation of cardiometabolic diseases, as they can improve different risk factors associated to plasma lipids, insulin resistance, hyperglycemia and inflammation (15). Furthermore, SCFA may have a beneficial impact on plasma TG levels through the modulation of key enzymes involved in lipid metabolism, leading to reduced TG levels (16). Concretely, it has been shown that dietary supplementation of acetate, propionate, butyrate or their admixture may modulate the expression of fatty acid receptors *FFAR2* and *FFAR3*, which ultimately may stimulate the hydrolysis of TG and enhance free fatty acid oxidization in adipose tissue (17). BAs, for their part, are involved in the regulation of multiple metabolic processes (18). Through the activation of various signaling pathways, BAs regulate not only their own synthesis and enterohepatic circulation, but also TG, cholesterol, glucose, and energy homeostasis (19). More concretely, BAs play a significant role in the digestion and absorption of fats, and may aid in the emulsification of dietary fats and fat-soluble vitamins, enhancing their absorption and regulating lipid metabolism, including TG levels.

The aim of the present study was to identify the metabolomic signature of responders and non-responders to n-3 FA supplementation, to test the potential effect and extent to which SCFAs and BAs may mediate changes in TG levels, and finally to test the ability of a multi-omic classifier combining genomics, lipidomics, and metabolomics to discriminate TG response phenotypes. We hypothesize that the metabolomic signature is likely to discriminate responders and non-responders to n-3 FA supplementation. These signatures may improve the predictive performance by applying a multi-omics classifier analysis combining genomic, lipidomic, and metabolomic features compared with exclusively genetic data.

## 2 Materials and methods

### 2.1 FAS study population

Between 2009 and 2011, a total of 254 healthy subjects living in the Quebec City metropolitan area were recruited to take part in the FAS Study. To be eligible, subjects must have not taken any n-3 FA supplements 6 months prior to the intervention, be non-smokers and have no thyroid or metabolic disorders requiring pharmacological treatment. Participants must have a body mass index (BMI) between 25 and 40 kg/m^2^ and be aged between 18 and 50. A total of 210 people completed the intervention protocol. Two participants were excluded from further analyses due to missing values prior to supplementation. Of the remaining 208 participants, those whose plasma TG levels decreased after the n-3 FA supplementation (ΔTG levels < 0) were defined as responders, while non-responders were participants for whom the TG concentrations remained stable or increased after the n-3 FA supplementation (ΔTG levels ≥ 0), as previously described (8). This study was approved by the ethics committees of Center de recherche du CHU de Québec and Université Laval and registered as NCT01343342 at ClinicalTrials.gov. All participants provided written informed consent prior to participation in accordance with the Declaration of Helsinki.

### 2.2 Study design

The study design and diets have been fully described previously (8). Briefly, after a 2-week stabilization period, the participants were asked to complete the intervention protocol, which consisted of consuming five capsules per day containing one gram of fish oil for a 6-week period. This supplementation provided 1.9–2.2 g of eicosapentaenoic acid (EPA) and 1.1 g of docohexaenoic acid (DHA) daily. Blood samples were taken immediately before and after n-3 FA supplementation, and plasma lipids were measured by enzymatic assays, as previously described (8).

### 2.3 Genomic, lipidomic, and metabolomic analyses

White blood cell samples were used for genomic analyses, while plasma was used for lipidomic and metabolomic analyses. First, the 141 participants who showed the most extreme plasma TG response to an n-3 FA supplementation were included in a previous GWAS (8) and its subsequent refinements (9). Briefly, the GenElute Gel Extraction Kit (Sigma-Aldrich Co.) was used to extract genomic DNA from the blood samples. Genotyping was conducted using the Illumina Human Omni-5 Quad BeadChip (Illumina, San Diego, CA), according to the manufacturer's instructions (8). After the exclusion of participants lacking plasma baseline samples, lipidomic analysis was performed on a total of 193 participants. As previously described in Picklo et al. (10), quantification of TG and CE species has been done using infusion mass spectrometry. Plasma levels of BA and SCFA were measured in 208 and 202 participants, respectively. Metabolomic analyses of SCFAs were carried out on the NUTRISS-INAF platform. Gas chromatography flame ionization detection (GC-FID) was used to profile plasma SCFA levels as previously described (20). Acetic, propionic and butyric acids were measured considering that they are the most abundant SCFAs, as well as isovaleric and isobutyric acids as the most abundant branched-chain fatty acids (BCFAs) (21). MS analyses were performed using electrospray ionization in the positive and negative ionization. Raw data were processed with Skyline (www.skyline.ms), Compound Discoverer 2.0 (Thermo Scientific, Waltham, MA, USA) and Progenesis QI QI v.2.1 (Non-linear Dynamics, Newcastle, UK). A calibration curve prepared with a mixture of standard SCFA and BCFA (acetic acid, propionic acid, isovaleric acid, butyric acid, isobutyric acid, and valeric acid) was used to measure these metabolites. An initial metabolite analysis was performed using the local reference standards database containing over 800 compounds (IROA technologies). The remaining chromatographic peaks were putatively identified using multiple spectral and molecular databases such as mzCloud and the Human Metabolome Database (HMDB), in addition to comparing retention time indices based on literature (22). Plasma levels of an exhaustive list of 25 primary and secondary BAs (23), as well as their glycine, taurine, sulfate, and glucuronide conjugates were measured. Concretely, plasma levels of chenodeoxycholic acid (CDCA), taurochenodeoxycholic acid (tauro-CDCA), glycochenodeoxycholic acid (glyco-CDCA), cholic acid (CA), taurocholic acid (tauro-CA), glycocholic acid (glyco-CA), ursodeoxycholic acid (UDCA), tauroursodeoxycholic acid (tauro-UDCA), lithocholic acid (LCA), taurolithocholic acid (tauro-LCA), glycolithocholic acid (glyco-LCA), deoxycholic acid (DCA), taurodeoxycholic acid (tauro-DCA), glycodeoxycholic acid (glyco-DCA), hyodeoxycholic acid (HDCA), hyocholic acid (HCA), LCA-3-sulfate, CDCA-3-glucuronide, CDCA-24-glucuronide, LCA-3-glucuronide, LCA-24-glucuronide, DCA-3-glucuronide, DCA-24-glucuronide, HDCA-6-glucuronide, and HCA-6-glucuronide were measured at the functional bileacidomic plateform by liquid chromatography/tandem mass spectrometry (LC-MS/MS), as previously described (24). The chromatographic system consisted of an Alliance 2690 HPLC instrument (Waters, Milford, MA), and the tandem mass spectrometry system (MS/MS) was an API4000 mass spectrometer (Applied Biosystems, Concord, Canada). A CDCA-3G standard curve was used to quantify HCA-6G, while all the other BA species were quantified with the appropriate standard curve (25).

### 2.4 Statistical analysis

A two-tailed unpaired *t*-test was used to compare baseline clinical characteristics and plasma metabolite levels between responders and non-responders. Linear mixed models, implemented in the lme (v3.1-162) and emmeans (v1.5.7) R packages, were used to test whether the changes in clinical outcomes and metabolomic markers during the intervention were significantly different between responders and non-responders. When a significant interaction at p<0.05 was found for any of the clinical parameters analyzed, contrasts analyses were performed to test its association with baseline levels of SCFA and BA in both responders and non-responders using emtrends (v1.8.4-1) and lmer (v1.1-31) R packages with adjustments for age, sex, batch and BMI, and age, sex, and BMI, respectively. A mediation analysis was conducted in the 141 participants with known genotypes, of which 66 were at high risk (GRS > median) and 75 at low risk (GRS ≤ median) of non-response to the supplementation. This analysis was carried out to examine the role of metabolomic markers as mediators in the relationship between baseline plasma TG levels and the change in plasma TG levels, depending on the levels of GRS values over the course of the intervention. The mediation analysis, which is comprised of three regression analyses, adjusted for age, sex, and BMI was performed with the mediation R package (v4.5.0). A mediation was considered significant at an Average Causal Mediation Effect (ACME) *p*-value < 0.05. Statistical analyses were performed with R [v4.1.2; (26)].

### 2.5 Predictive analysis

Different classifiers aiming at accurately identifying responders and non-responders to the n-3 FA supplementation, and combining genomic, lipidomic, and metabolomic features were developed. The classification tools were built and tested within the DIABLO (Data Integration Analysis for Biomarker Discovery Using Latent Components) framework, which implements a partial least squares-discriminant analysis (PLS-DA) to multi-omics analysis (27) in the mix0mics R package (v6.20.0) (28). The classifiers were first tuned in the entire sample independently for each predictor and its performance was evaluated with the area under the ROC curve (AUC-ROC) and the balanced accuracy metrics. The sample was then randomly divided into two data sets, one for training and the other for testing, maintaining the same proportions of responders and non-responders. To reach the highest prediction value, different classifiers were developed by combining data from genomics (31 SNPs), lipidomics (13 cholesterol esters and 57 TG species) and metabolomics (6 SCFA and 25 BA species). Each classifier was then trained and a 10-fold cross-validation was performed using the R caret package (v6.0-94) (29). The predictive performance of cross-validated models obtained in the train dataset was further assessed in the test dataset. Predictive performance comparison between classifiers was finally evaluated in the test dataset using the balanced accuracy metric, which stands for the proportion of true responders and non-responders out of the total number of subjects.

## 3 Results

### 3.1 Characteristics of the study participants

Clinical characteristics of study participants in pre- and post-supplementation have been previously reported in Rudkowska et al. (8) and are briefly summarized herein. Participants were overweight, with a mean BMI of 27.8 ± 0.3 kg/m^2^ for the responders and 27.8 ± 0.5 kg/m^2^ for the non-responders. Although body weight increased in both responders and non-responders following the intervention, it was significantly higher in responders (*p*_group*visit_ = 0.05), also reflected in BMI (*p*_group*visit_ = 0.04). Following the intervention, responders showed a mean decrease in insulin levels of 9.8%, while non-responders showed an increase of 11.6% (*p*_group*visit_ = 0.06, Table 1). Responders had significantly higher plasma TG levels before the supplementation than non-responders (1.28 ± 0.05 vs. 1.03 ± 0.08 mmol/L; *p* < 0.01), and a significant group-by-visit interaction effect was found for plasma TG levels (*p*_group*visit_ < 0.01; Table 1).

**Table 1:** Clinical parameters of responders and non-responders during the n-3 fatty acid supplementation.

	**Responders (*****n*** = **148)**		**Non-responders (*****n*** = **60)**		***P*** **-values**		
**Variables**	**Pre**	**Post**	**Pre**	**Post**	**Group**	**Time**	**Int**
Sex (men/women)	66/82		30/30		0.58	-	-
Age (years)	30.7 ± 8.6		31.1 ± 9.0		0.77	-	-
Weight (kg)	80.8 ± 1.1	81.0 ± 1.1	82.6 ± 1.8	83.2 ± 1.8	0.23	0.05	0.15
BMI (kg/m^2^)	27.8 ± 0.3	27.9 ± 0.3	27.8 ± 0.5	28.0 ± 0.5	1.00	<0.01	**0.04**
Glucose (mmol/L)	4.96 ± 0.04	5.06 ± 0.04	4.94 ± 0.06	5.04 ± 0.06	0.58	<0.01	0.96
Insulin (pmol/L)	90.0 ± 5.9	81.2 ± 3.3	80.2 ± 9.3	89.5 ± 5.3	0.13	0.32	0.06
Total-C (mmol/L)	4.74 ± 0.07	4.66 ± 0.08	4.77 ± 0.12	4.87 ± 0.12	0.47	0.41	**0.02**
TG (mmol/L)	1.28 ± 0.05	0.95 ± 0.04	1.03 ± 0.08	1.20 ± 0.07	<0.01	<0.01	**<0.01**
HDL-C (mmol/L)	1.41 ± 0.03	1.47 ± 0.03	1.50 ± 0.05	1.48 ± 0.05	0.03	<0.01	**0.01**
LDL-C (mmol/L)	2.75 ± 0.07	2.76 ± 0.07	2.79 ± 0.11	2.84 ± 0.11	0.75	0.47	0.67
Total-C/HDL-C (mmol/L)	3.55 ± 0.09	3.40 ± 0.09	3.33 ± 0.13	3.48 ± 0.14	0.11	0.01	**<0.01**
ApoB (g/L)	0.84 ± 0.02	0.86 ± 0.02	0.83 ± 0.03	0.89 ± 0.03	0.85	<0.01	0.08
CRP (mg/L)	2.47 ± 0.32	2.50 ± 0.36	2.89 ± 0.51	3.06 ± 0.56	0.22	0.85	0.87
Folic acid (nmol/L)	35.5 ± 0.6	34.9 ± 0.5	34.7 ± 0.9	31.6 ± 0.9	0.02	<0.01	**0.01**
B12 vitamin (mcg)	328.6 ± 10.3	341.8 ± 10.7	344.0 ± 16.2	335.3 ± 16.8	0.74	0.06	**0.01**
Total bilirubin (pg/L)	9.67 ± 0.46	9.78 ± 0.56	11.29 ± 0.71	10.88 ± 0.87	0.07	0.96	0.43
Direct bilirubin (pg/L)	2.32 ± 0.09	2.38 ± 0.09	2.69 ± 0.14	2.45 ± 0.14	0.15	0.75	**0.03**
Indirect bilirubin (pg/L)	7.35 ± 0.38	7.39 ± 0.49	8.60 ± 0.59	8.43 ±0.75	0.10	0.99	0.75

Data are unadjusted means ± standard deviation. A linear mixed model adjusted for age, sex, and body mass index (BMI) was used to compare clinical parameters between responders and non-responders to the n-3 fatty acid supplementation. A linear mixed model adjusted for age and sex was used to compare BMI.

Total-C, total cholesterol; HDL-C, HDL-cholesterol; LDL-C, LDL-cholesterol; ApoB, apolipoprotein B; CRP, C-reactive protein; Pre, pre-supplementation values; Post, post-supplementation values. Int, group-by-time interaction term. Bold values represent significant associations *p* < 0.05.

### 3.2 Metabolomic analysis

**Figure 1:**
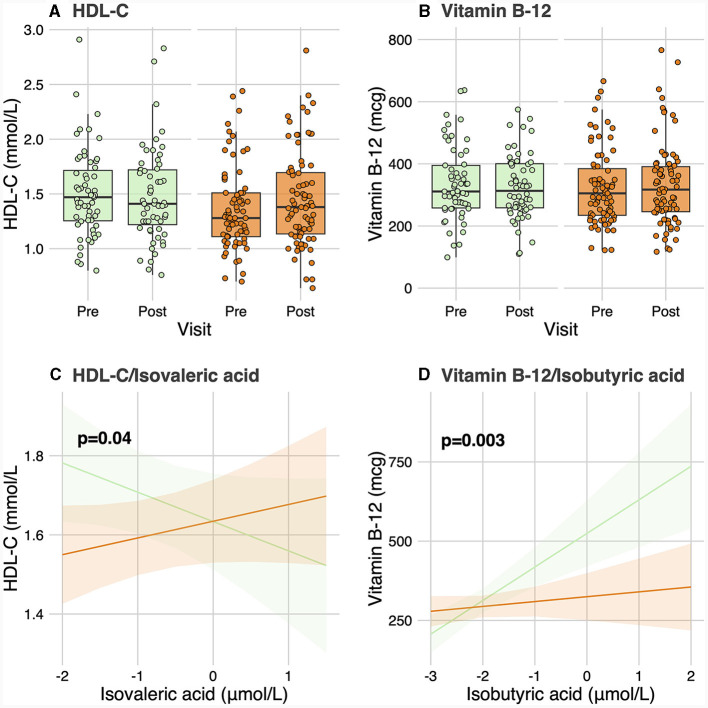
Contrast analysis between short-chain fatty acid levels and clinical parameters. **(A, B)** show levels of HDL-C and vitamin B-12, respectively, for responders and non-responders before and after the intervention. **(C)** shows the change in HDL-C levels related to the change in isovaleric acid levels for responders and non-responders. **(D)** shows the change of vitamin B-12 linked to the change in isobutyric acid levels for both groups. HDL-C, High-density lipoprotein cholesterol.

No significant differences were observed in baseline levels for any of the 6 SCFA and 25 BA analyzed between responders and non-responders (Table 2). No significant group-by-visit interaction effects were found for acetic acid, propionic acid, isobutyric acid, butyric acid, isovaleric acid, or valeric acid (Table 2). No significant group-by-visit interaction effects were observed for any of the BAs analyzed either (Table 2). Contrast analyses were performed between baseline plasma SCFA levels and clinical parameters with significant group-by-visit interaction effects during the intervention (Table 1). Pre- and post-supplementation levels of the two clinical parameters having a significant contrast effect with SCFA, HDL-C (*p*_group*visit_ = 0.01, Figure 1A) and vitamin B-12 (*p*_group*visit_ = 0.01, Figure 1B) are shown. A significant association was found between HDL-C and isovaleric acid (*p* = 0.04; Figure 1C), with a positive association between HDL-C and isovaleric acid in responders, and a negative association in non-responders (Figure 1C). A significant association was also found between vitamin B-12 and isobutyric acid (*p* = 0.003; Figure 1D). Along the same lines, contrast analyses performed between baseline plasma BA levels and the change in clinical parameters during the intervention showed a significant association between HDL-C and tauro-CA (*p* = 0.009). A negative association between plasma HDL-C and tauro-CA levels in responders was found, while the opposite was observed for non-responders, i.e., a positive association between the increase in HDL-C and in tauro-CA level was found. Significant associations between total-C and the sum of tauro-conjugated bile acids (*p* = 0.04), tauro-CA (*p* = 0.006), and glyco-CA (*p* = 0.02) were also found.

**Table 2:** Metabolomic analysis of short-chain fatty acid and bile acid levels of responders and non-responders during the n-3 fatty acid supplementation.

	**Responders**		**Non-responders**		***P*** **-values**		
**Variables**	**Pre**	**Post**	**Pre**	**Post**	**Group**	**Time**	**Int**
**Short-chain fatty acids**							
Acetic acid	49.69 ± 1.53	50.39 ± 1.53	50.96 ± 2.38	48.82 ± 2.38	0.72	0.99	0.38
Propionic acid	1.06 ± 0.04	1.07 ± 0.04	1.06 ± 0.06	1.12 ± 0.06	0.68	0.30	0.83
Isobutyric acid	0.26 ± 0.02	0.27 ± 0.02	0.25 ± 0.03	0.25 ± 0.03	0.81	0.46	0.79
Butyric acid	0.49 ± 0.02	0.50 ± 0.02	0.53 ± 0.03	0.57 ± 0.03	0.26	0.28	0.95
Isovaleric acid	0.63 ± 0.02	0.66 ± 0.02	0.64 ± 0.03	0.63 ± 0.03	0.86	0.65	0.21
Valeric acid	0.10 ± <0.01	0.10 ± <0.01	0.10 ± <0.01	0.10 ± <0.01	0.54	0.95	0.38
**Bile acids**							
CDCA	3.61 ± 0.89	6.39 ± 0.92	2.71 ± 1.48	3.56 ± 1.34	0.46	0.26	0.33
Tauro-CDCA	3.74 ± 0.85	4.73 ± 0.88	2.01 ± 1.30	4.42 ± 1.19	0.19	0.34	0.84
Glyco-CDCA	3.26 ± 0.50	2.25 ± 0.51	1.60 ± 0.73	2.77 ± 0.68	0.88	0.17	0.73
CA	9.32 ± 1.88	9.49 ± 1.95	6.87 ± 3.08	7.82 ± 2.80	0.66	0.85	0.80
Tauro-CA	4.24 ± 2.48	8.40 ± 2.54	4.26 ± 3.47	4.74 ± 3.22	0.20	0.05	0.90
Glyco-CA	4.16 ± 0.79	3.25 ± 0.81	1.70 ± 1.14	3.62 ± 1.06	0.30	0.73	0.82
UDCA	3.05 ± 1.08	5.75 ± 1.12	1.99 ± 1.76	2.68 ± 1.61	0.80	0.29	0.67
Tauro-UDCA	8.97 ± 2.78	10.73 ± 2.91	6.48 ± 4.77	6.81 ± 4.28	0.41	0.08	0.70
ICA	1.45 ± 0.14	1.59 ± 0.14	1.41 ± 0.21	1.45 ± 0.19	0.99	0.15	0.12
Tauro-LCA	3.29 ± 0.71	3.11 ± 0.74	3.01 ± 1.20	3.70 ± 1.09	0.67	0.37	0.49
Glyco-LCA	4.07 ± 0.84	2.59 ± 0.85	2.87 ± 1.03	3.54 ± 0.98	0.08	0.91	0.71
LCA sulfate	5.01 ± 1.46	3.62 ± 1.47	8.22 ± 2.45	8.72 ± 2.25	0.06	0.61	0.93
DCA	1.59 ± 0.19	1.85 ± 0.20	1.61 ± 0.31	1.23 ± 0.28	0.61	0.62	0.14
Tauro-DCA	4.56 ± 1.17	5.57 ± 1.21	3.09 ± 1.83	3.73 ± 1.66	0.27	0.64	0.26
Glyco-DCA	3.77 ± 0.72	3.16 ± 0.73	1.89 ± 0.93	3.22 ± 0.87	0.65	0.97	0.90
HDCA	1.68 ± 0.14	1.47 ± 0.14	1.71 ± 0.22	1.41 ± 0.20	0.31	0.98	0.43
HCA	2.13 ± 0.31	2.31 ± 0.32	1.95 ± 0.51	2.11 ± 0.46	0.67	0.23	1.00
CDCA-3-G	2.00 ± 0.25	1.94 ± 0.25	1.98 ± 0.35	2.05 ± 0.32	0.62	0.75	0.73
CDCA-24-G	4.05 ± 0.76	4.78 ± 0.79	3.06 ± 1.33	5.14 ± 1.18	0.79	0.69	0.99
LCA-3-G	2.56 ± 0.24	2.03 ± 0.25	2.56 ± 0.38	2.13 ± 0.34	0.61	0.02	0.18
LCA-24-G	1.55 ± 0.43	3.06 ± 0.45	1.48 ± 0.70	3.41 ± 0.63	0.09	<0.01	0.71
DCA-3-G	1.95 ± 0.20	1.83 ± 0.20	2.66 ± 0.30	1.58 ± 0.27	0.99	0.03	0.24
DCA-24-G	1.39 ± 0.14	1.64 ± 0.14	1.65 ± 0.21	1.32 ± 0.19	0.96	0.37	0.26
HDCA-6-G	1.53 ± 0.14	1.32 ± 0.15	1.44 ± 0.23	1.48 ± 0.21	0.33	0.03	0.94
HCA-6-G	2.27 ± 0.28	1.77 ± 0.28	2.16 ± 0.42	2.47 ± 0.39	0.39	0.80	0.89

Data are unadjusted means ± standard deviation. A linear mixed model adjusted for batch, age, sex and BMI was used to compare plasma SCFA levels between responders and non-responders to the n-3 fatty acid supplementation. A linear mixed model adjusted for batch, age, sex, and BMI was used to compare plasma SCFA levels.

CDCA, chenodeoxycholic acid; Tauro-CDCA, taurochenodeoxycholic acid; Glyco-CDCA, glycochenodeoxycholic acid; CA, Cholic acid; Tauro-CA, taurocholic acid; Glyco-CA, glycocholic acid; UDCA, Ursodeoxycholic acid; Tauro-UDCA, tauroursodeoxycholic acid; LCA, lithocholic acid; Tauro-LCA, taurolithocholic acid; Glyco-LCA, glycolithocholic acid; DCA, deoxycholic acid; Tauro-DCA, taurodeoxycholic acid; Glyco-DCA, glycodeoxycholic acid; HDCA, hyodeoxycholic acid; HCA, hyocholic acid; −3, −6 and 24G, 3, 6 and 24-glucuronide; Pre, pre-supplementation values; Post, post-supplementation values. Int, group-by-time interaction term.

N for short-chain fatty acids = 202 and N for bile acids = 208.

### 3.3 Mediation analysis

Previously, baseline plasma TG levels and GRS were identified as the main factors leading to the heterogeneity of plasma TG response to an n-3 FA supplementation (9). The role of SCFAs as mediators in the different TG response to n-3 FA between responders and non-responders was examined through a mediation analysis. The significant association found between baseline plasma TG levels and the change in plasma TG levels during the supplementation was found to be partially mediated by baseline plasma levels of isovaleric acid in participants having a high genetic risk (GRS > median) of non-response (ACME *p*-value = 0.04). This significant association vanished when tested in participants with a low genetic risk (GRS <median) of non-response (ACME *p*-value = 0.8). The role of BAs as mediators in this relationship was also examined and no significant mediation effect was found in any of the high or low genetic risk subgroups.

### 3.4 Predictive analysis

**Figure 2:**
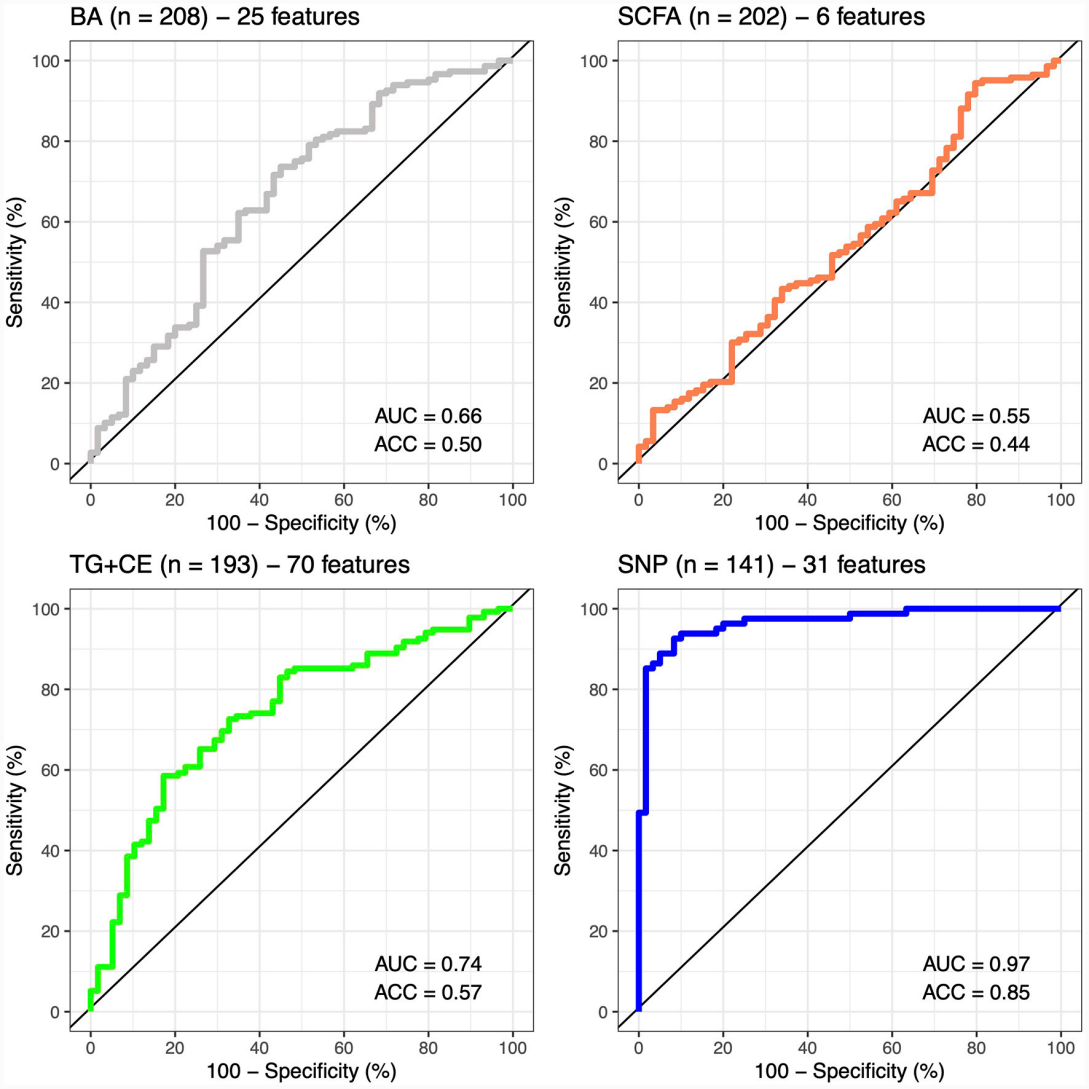
Predictive performance of metabolomic, lipidomic, and genomic features. The predictive performance of each feature is represented by the area under the ROC-curve (AUC) and the balance accuracy (ACC) metrics. AUC, area under the curve; ACC, accuracy; BA, bile acids; SCFA, short-chain fatty acids; TG + CE, triglycerides and cholesterol esters; SNP, single nucleotide polymorphism.

**Figure 3:**
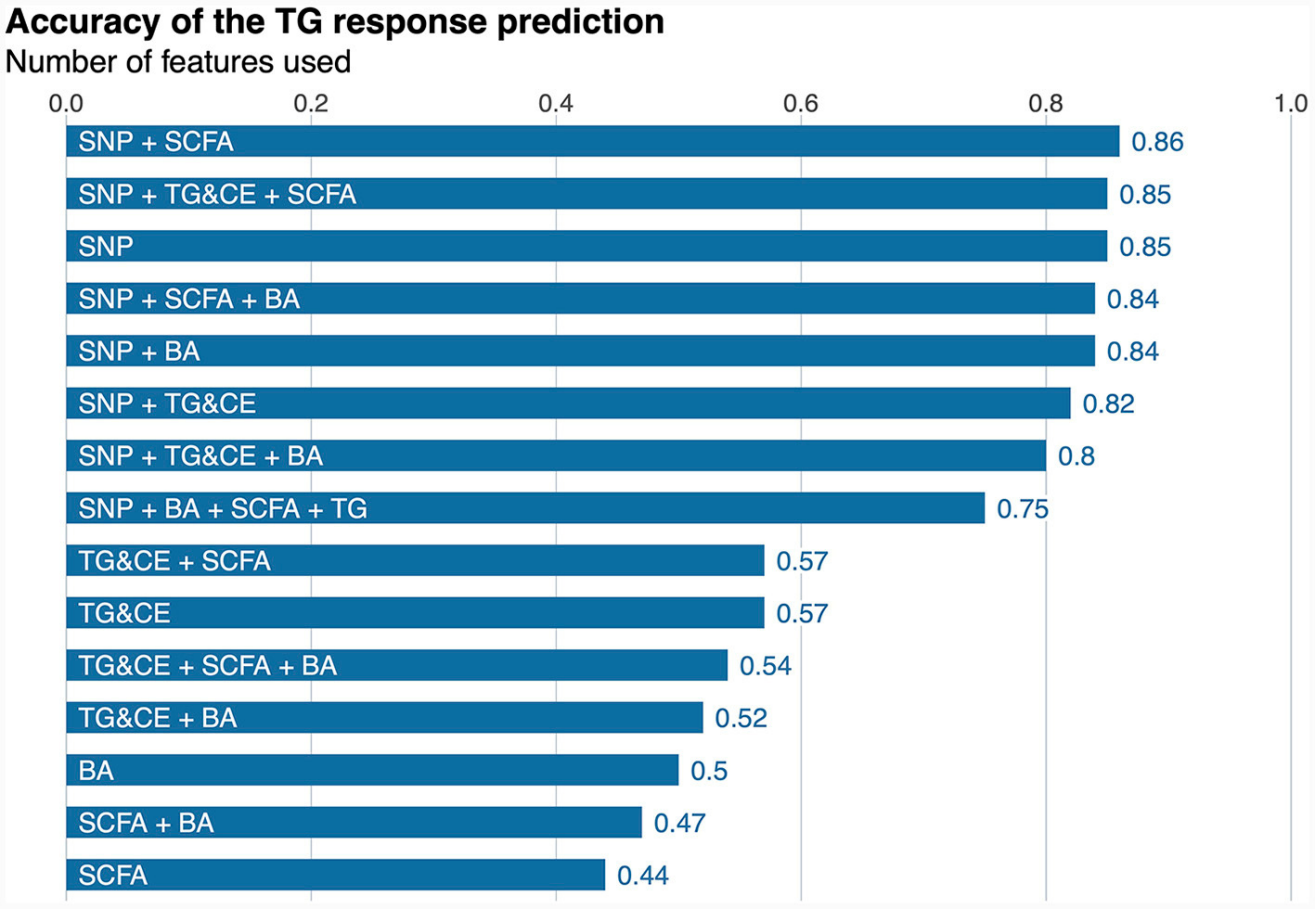
Predictive performance of the response to an n-3 FAs supplementation. SNP, single nucleotide polymorphism; SCFA, short-chain fatty acid; TG&CE, triglycerides and cholesterol esters; BA, bile acids; n-3 FA, omega-3 fatty acid. The balance accuracy for each feature or combination of features is shown the top of the bars.

A classifier was built independently for each feature to separately assess the ability of genomic, lipidomic, and metabolomic data to accurately predict response to the n-3 FA supplementation. The classifier containing only baseline plasma SCFAs achieved the lowest predictive performance, with an AUC-ROC of 0.5 and an accuracy of 44% (Figure 2), whereas the one created exclusively with baseline plasma BAs only reached an AUC-ROC of 0.66 and a balanced accuracy of 50% (Figure 2). The classification tool built using uniquely lipidomic features slightly increased the AUC-ROC to 0.74 and the accuracy to 57% (Figure 2), while the use of genomic data alone made it possible to create the best independent classifier, with a predictive performance reaching an AUC-ROC of 0.97 and a balanced accuracy of 85% (Figure 2). After testing for each feature individually, genomic, lipidomic and metabolomic data layers were combined to increase the predictive performance of the response to an n-3 FA supplementation. In the test dataset, we observed that none of the combined classifiers was able to substantially increase the predictive performance showed by genomic data alone (Figure 3). Only a classifier built by adding plasma SCFAs and lipidomic data to the 31 SNPs was able to achieve the same accuracy of 85% shown by the genomic predictor (Figure 3). Interestingly, the classifier built by combining plasma SCFAs and genomic data slightly increased the predictive performance to a balanced accuracy of 86% (Figure 3). The predictive capacity of the complete four-layer-model, including the 31 SNPs and lipidomic data (13 cholesterol esters and 57 TG species), as well as 6 SCFA and 25 BA species, reached an overall balanced accuracy of 75% (Figure 3). Then, the predictive performance was refined by further selecting the most important features on each layer. After a tuning process involving a 10-fold-cross validation and based on the centroids distance, we finally kept a reduced model including 28 SNPs as well as 10 TG, 2 SCFA and 5 BA species. However, the accuracy of the refined classification tool only reached 76%.

## 4 Discussion

The present study examined the role of SCFAs and BAs in the heterogeneity of the TG response to an n-3 FA supplementation. A continually emerging body of evidence supports the role of SCFAs and BAs as key molecular links between diet, the microbiome and health (30). Thus, in the absence of data on the gut microbiota, it is possible to indirectly analyze the impact of gut microbiota composition on the heterogeneity observed in the metabolic response to n-3 FA supplementation through the metabolites it produces. The present study did not reveal that plasma levels of SCFA and BA play a relevant role in the clinical and metabolic changes observed following an n-3 FA supplementation, and they lack of predictive power to accurately classify responders and non-responders to the supplementation. Indeed, results showed that SCFAs but not BAs were marginally relevant in the classification of plasma TG responses. In this regard, genomic data still represent the best predictor of plasma TG response to an n-3 FA supplementation (9), and the inclusion of other omic data layers does not substantially increase the ability to discriminate the plasma TG response phenotypes.

As previously stated, the consumption of specific nutritional components can modify the abundance, diversity, and activity of the gut microbiota, and consequently the composition in SCFAs and BAs (31). Although this may have been observed in the present study, no significant differences between responders and non-responders were found in plasma levels during the n-3 FA supplementation for any of the SCFAs and BAs analyzed. However, the previously reported significant association between baseline plasma TG levels and change in plasma TG levels in response to n-3 FA supplementation (9) was here partially mediated by plasma isovaleric acid levels at baseline. Both baseline plasma TG levels and GRS have been previously identified as the main factors leading to the plasma TG response heterogeneity to an n-3 FA supplementation (9). In the present study, the mediating effect of isovaleric acid was reported between baseline TG levels and response to n-3 FA supplementation in the group of participants at high genetic risk of non-response. In other words, the relationship between basal TG levels and response to n-3 FA supplementation appears to be partially mediated by isovaleric acid when GRS is high. A significant association was also found between baseline plasma isovaleric acid levels and the change in HDL-C levels during the intervention. Concretely, plasma HDL-C levels increased together with isovaleric acid levels in the group of responders, whereas in non-responders this relationship was inverse. High plasma HDL-C levels act to reduce cardiovascular risk by multiple pathways (32). Therefore, in non-responders, an increase in isovaleric acid levels would have a negative effect on cardiovascular risk, as plasma HDL-C levels decrease. Consistently, a previous study found a positive correlation between isovaleric acid and HDL-C, as well as positive correlations between isovaleric acid and LDL-C and total-C in pregnant women (33). The significant association between isovaleric acid and the change in plasma HDL-C levels found in the present study is also in agreement with recent findings showing a positive correlation between isovaleric acid and LDL-C levels, as well as between valeric acid and TG levels, and between isobutyric acid and HDL-C and LDL-C levels (34). An increase in BCFA levels has been also observed in individuals with hypercholesterolemia, as compared to those with normocholesterolemia (35), suggesting a potential association between BCFAs and lipid metabolism. *In vitro* experiments have also shown that BCFAs are able to inhibit both cAMP-mediated lipolysis and insulin-stimulated lipogenesis in adipocytes (36). Moreover, isovaleric acid-containing porpoise oil has shown to exert beneficial effects on fatty liver in a murine model of type 2 diabetes, by increasing serum levels of adiponectin and enhancing lipoprotein synthesis and secretion (37).

The synergistic role of n-3 FA and vitamin B-12 in lipid homeostasis and other host metabolic processes has been extensively studied in rats. Concretely, a series of studies showed that n-3 FA are linked with vitamin B-12 in the one-carbon metabolic cycle (38). A previous study also reported that maternal vitamin B-12 deficiency in pregnant Wistar rats resulted in elevated homocysteine levels, a vitamin B-12 substrate, while the supplementation with n-3 FA reduced homocysteine levels (39). As these animals were deliberately induced into a vitamin B-12 deficient state, these studies did not find an increase in vitamin B-12 after the n-3 FA supplementation, but have already highlighted a common metabolic pathway that may explain why responders to n-3 FA supplementation also showed increased vitamin B-12 levels in the present study. Furthermore, by modulating the gut microbiota composition, n-3 FA may indirectly affect vitamin B-12 absorption or production differently in responders and non-responders. Most dietary vitamin B-12 in humans comes from animal-derived foods, and is absorbed in the small intestine. However, the presence of vitamin B12-producing bacteria in the colon may contribute to the B-12 supply (40). The role of gut microbiota in contributing to vitamin B-12 status remains an area of ongoing research, exploring the impact of different bacterial strains on host vitamin B-12 metabolism. In this regard, the significant association observed between vitamin B-12 and isobutyric acid may potentially be explained by the protein composition of the diet. Indeed, high protein diets have been related with higher BCFAs levels (41, 42) and as widely recognized, vitamin B-12 is bound to protein in food (43). We can also speculate that n-3 FA may act as a prebiotic and indirectly affect the production of vitamin B-12 and isobutyric acid differently among responders and non-responders, through a distinct modulation of gut microbiota composition. In this regard, a previous study has shown an increase in the production of vitamin B-12, along with SCFA and BCFA, including isobutyric acid, during the *in vitro* digestion and fermentation of probiotic chocolate (44), which may be attributed to shared *Lactobacillus* species.

Another significant association was found between the change in plasma HDL-C levels during the intervention and tauro-CA levels. Specifically, a negative association was found between plasma HDL-C levels and tauro-CA levels in the group of responders, whereas a positive association was seen for non-responders. The significant association found between HDL-C and tauro-CA is in line with the negative relationship between plasma levels of HDL-C and biliary saturation previously reported in healthy females (45). This observation can be explained by the fact that HDL-C has been proposed to serve as preferential precursor for BA biosynthesis (45). McMillin and al. found that the three ratios of taurine-conjugated BAs (primary or secondary) and the levels of tauro-UDCA and tauro-HDCA were elevated following a fish oil supplementation. These results indicate that n-3 FAs may promote taurine conjugation with BAs or inhibit the deconjugation of taurine-conjugated BAs (46). All associations observed in the present study between SCFAs and BAs with cholesterol levels thus suggest a potential role of gut microbiota in lipid homeostasis. Current research is gradually enabling us to understand how the gut microbiota influences cholesterol metabolism in order to eventually target it for therapeutic benefit (47).

Gut microbiota speciation has proven to have a regulatory role in host lipid metabolism (48), and specifically in plasma TG and cholesterol levels, as reviewed in Schoeler and Caesar (49). Hence, it has been proposed that gut microbiota may be key to the development of hyperlipidemia and related chronic diseases, such as cardiovascular disease (14). In this regard, previous studies with conventionally raised and germ-free mice have elucidated that the gut microbiota prompts hepatic fatty acid synthesis (50). Similarly, it has been shown that the interaction between gut microbiota with dietary components may impact host lipid metabolism and composition. Concretely, results from mice fed diets rich in saturated lipids supplemented with BA revealed that the gut microbiota was involved in the observed increase of hepatic TG levels, highlighting the influence of colonization status on hepatic lipid profiles (51). Additionally, research involving probiotic treatments in mice and rats further underscores the role of the gut microbiota in regulating lipid homeostasis. For instance, in mice fed a high-fat high-cholesterol diet, *Lactobacillus curvatus* alone or in combination with *Lactobacillus plantarum* significantly reduced cholesterol levels in plasma and liver, while exhibiting a synergistic effect on hepatic TG levels (52). Similarly, rats on a high-fat diet experienced decreased circulating TG and LDL levels, along with increased HDL levels, when administered *Bifidobacterium* spp. (53). Moreover, clinical studies connecting the gut microbiota to lipid metabolism have revealed that reduced microbial gene richness in individuals with obesity correlated with higher plasma total-cholesterol and TG levels (54). An energy-restricted diet has also been shown to increase gene richness and reduce lipid levels. Individuals with fewer microbial had higher TG levels and lower HDL-cholesterol levels (55). Additionally, variability in gut microbiota composition have been associated with about 6% of plasma TG levels and 4% of HDL-cholesterol levels in the general population (56).

Although genetic and lipidomic factors have been reported to cover a significant portion of the variance in the plasma TG response to an n-3 FA supplementation, a substantial fraction of this response remains unexplained. This still unexplained variability may be influenced by several other factors, such as dietary habits, including fat and carbohydrate intake, as well as specific nutrients that may interact with or modify the effects of n-3 FA. A role of individual metabolism and absorption of n-3 FA due to differences in microbial and host enzymatic activity is another potential factor leading to this heterogeneity, as well as the changes in gut microbiota composition investigated in this study. Nevertheless, the present results did not support the use of baseline SCFA and BA levels as a proxy of gut microbiota composition in the prediction of the interindividual variability in the plasma TG response to n-3 FA supplementation. In this sense, it is worth highlighting that, even in the absence of direct measurements of gut microbiota, this study still has some strengths. Multiple clinical and metabolomic measurements were carried out, which adds to the power of the study, as well as the thoughtful assessment of the compliance of participants to the intervention protocol. However, the fact that this study was conducted on a mostly Caucasian population living in the Quebec City metropolitan area limits the generalization of the results obtained, as well as the relatively small sample size. Moreover, it has been observed that the higher the baseline levels of TG, the more effective the n-3 FA supplementation is in reducing TG levels (9). However, since one of the exclusion criteria is having a metabolic disorder, participants who had higher baseline TG levels were not included in the study. On the one hand, this may have influenced the results, but on the other hand, it makes it possible to generalize the results to a healthy population.

In the FAS Study, participants were classified as responders and non-responders based on changes in plasma TG levels subsequent to an n-3 FA supplementation. This categorization approach, although providing a simplified interpretation of outcomes, presented several limitations warranting discussion. First, the binary classification into responders and non-responders may have overlooked the inherent variability within these groups. Quantitative phenotypes may have offered a more nuanced understanding, capturing the spectrum of individual responses to the supplementation. Second, by adopting a categorical approach, we acknowledge the potential oversimplification of complex physiological responses, potentially masking variations in treatment effects within these broad categories. However, we have previously shown that the classification of participants into responders and non-responders constituted a major strength of the FAS Study, allowing a growing reliability in the identification of actual responders by refining the predictive models (8–10, 57, 58). In terms of clinical relevance, we recently reported that a prognostic model with such a high predictive performance may be a suitable decision aid tool to identify individuals likely to benefit from n-3 FA supplementation in reducing plasma TG levels (57). Nevertheless, such a predictive tool would need to be validated first in a larger and heterogeneous cohort to be able to guide treatment choices, and should emphasize sensitivity (identifying actual responders) over specificity (accurately classifying non-responders) to maximize the number of patients benefiting from n-3 FA treatment. In any case, integrating quantitative phenotypes may yield a more comprehensive insight into the magnitude and patterns of response, facilitating a more precise interpretation of the supplementation efficacy and aiding in targeted interventions. Thus, future studies should explore the continuum of responses to provide a deeper understanding of individual variations in the context of n-3 FA supplementation and plasma TG modulation.

Both the host phenotype and genotype, as well as other geographical and environmental factors, may influence the microbial composition in taxa type and abundance (59). More precisely, it was found that as much as 20% of the microbiota variability was associated with diet, medication and body composition (60). In any case, an in-depth study of the gut microbiota is limited by the absence of fecal samples. Thus, future studies are still needed to determine its role on the metabolic heterogeneity observed in response to food consumption, as well as the impact of diet on its composition, diversity and activity. Finally, the complex interaction between genetics, environment, lifestyle, and individual health characteristics also contribute to the heterogeneity in the response to a nutritional intervention. Exploring these factors through further research may help elucidate the unrevealed variability in the plasma TG response to an n-3 FA supplementation.

## 5 Conclusion

In conclusion, identifying the underlying factors of the interindividual variability observed in the metabolic response to n-3 FA will make it possible to develop precision tools to better prevent chronic societal diseases by modifying lifestyle habits, including diet. In this study, metabolomic changes resulted from n-3 FA supplementation showed a limited impact on the metabolic response derived from it, as well as on the predictive ability of this response. However, the integration of multi-omics data still represents a promising approach to cardiovascular disease prevention, and further efforts including more comprehensive data, for example intestinal metagenomics, may help to elucidate the yet unexplained inter-individual variability observed in the metabolic response to n-3 FA supplementation.

## Data availability statement

The raw data supporting the conclusions of this article will be made available by the authors, without undue reservation.

## Ethics statement

The studies involving humans were approved by Center de recherche du Centre hospitalier universitaire de Québec and Université Laval. The studies were conducted in accordance with the local legislation and institutional requirements. The participants provided their written informed consent to participate in this study.

## Author contributions

JM-B: Data curation, Formal analysis, Methodology, Writing – original draft, Writing – review & editing. JT-M: Data curation, Formal analysis, Methodology, Writing – review & editing. VB: Data curation, Formal analysis, Methodology, Writing – review & editing. RS-C: Data curation, Formal analysis, Methodology, Writing – review & editing. SL: Writing – review & editing. IR: Writing – review & editing. PC: Writing – review & editing. OB: Writing – review & editing. M-CV: Supervision, Writing – review & editing.
