# Corrigendum: Revisiting multi-omics-based predictors of the plasma triglyceride response to an omega-3 fatty acid supplementation

**DOI:** 10.3389/fnut.2024.1388485

**Published:** 2024-03-08

**Authors:** Josiane Morin-Bernier, Juan de Toro-Martín, Valentin Barbe, Rodrigo San-Cristobal, Simone Lemieux, Iwona Rudkowska, Patrick Couture, Olivier Barbier, Marie-Claude Vohl

**Affiliations:** ^1^Centre Nutrition, santé et société (NUTRISS)—Institut sur la nutrition et les aliments fonctionnels (INAF), Université Laval, Québec, QC, Canada; ^2^School of Nutrition, Université Laval, Québec, QC, Canada; ^3^Laboratory of Molecular Pharmacology, Endocrinology and Nephrology Unit, Centre hospitalier universitaire de Québec-Université Laval Research Center, Québec, QC, Canada; ^4^Department of Kinesiology, Université Laval, Québec, QC, Canada; ^5^Faculty of Pharmacy, Université Laval, Québec, QC, Canada

**Keywords:** short-chain fatty acids, bile acids, gut microbiota, metabolic health, metabolomics, precision nutrition

In the published article, there was an error in the description of the measurements of the short-chain fatty acids (SCFA).

A correction has been made to Materials and methods, *2.3 Genomic, lipidomic and metabolomic analyses*. This sentence previously stated:

“Metabolomic analyses of SCFAs were carried out on the NUTRISS-INAF platform. Ultra-high performance liquid chromatography coupled to mass spectrometry (U-HPLC-MS) was used to profile plasma SCFA levels.”

The corrected sentence appears below:

“Metabolomic analyses of SCFAs were carried out on the NUTRISS-INAF platform. Gas chromatography flame ionization detection (GC-FID) was used to profile plasma SCFA levels as previously described (1).”

The authors apologize for this error and state that this does not change the scientific conclusions of the article in any way. The original article has been updated.
